# Chorea and Its Treatment

**Published:** 1905-11-25

**Authors:** 


### CHOREA AND ITS TREATMENT.

A PAPER by Dr. H. Grenet [1] on the treatment of chorea serves to remind us of one of the many mysteries which still surround this common and dramatic disease. How far is chorea a neurosis, and how far an expression of organic departures from the normal? What is the true relation of chorea and rheumatism when they concur in the same patient at different times, and what is to be thought of those cases in which no relation whatever appears possible of establishment? Such questions as these, to which none but vague answers are forthcoming, point the uncertainties of our present position with regard to chorea. It may, however, be of value to review the opinions of some authoritative writers on the problems stated above before proceeding with Dr. Grenet's suggestions for treatment.

It is premised that the disease in question is Sydenham's chorea, a malady totally distinct from the many forms of habit-spasm or athetosis which distantly simulate it. The prime distinction lies in the fact that in the case of tics the same movement is constantly repeated, while in chorea proper there is no such limitation and regularity.

With regard, then, to the functional or organic nature of chorea, Dr. Goodhart, after traversing the old and fascinating explanation which credited the movements of chorea to minute embolisms of cerebral arteries by fragments of the vegetations not infrequently formed upon the mitral and aortic valves, delivers himself as follows: "It seems to me that a study of this disease leads to the conclusion that it is one unassociated with any recognisable structural change in the nervous system—that it is, in fact, a functional disease. We see this in the antecedents of the child, both parental and individual, we see it in the disease itself, in the want of control, in the emotional excitement, in some cases in its relationship to hysteria, and in its all but certain tendency towards cure. . . What the relation of rheumatism to chorea may be we do not know; but for my part I believe that the rheumatic taint, whatever that may be, points out the individual in whom it exists as one in whom various morbid nervous phenomena are likely to show themselves, whose nerve-textures, cerebral more particularly, are easily impoverished, and, being inherently bad or easily exhausted, discharge intermittently, erratically, and feebly." Subsequently this author lays stress upon the fact that very frequently chorea is the first evidence of rheumatism, the franker indications of this malady being later in their appearance. This position is supported by an investigation of Dr. Batten, which resulted as follows. Tabulation of 115 cases of chorea showed that 32 per cent. had previously had rheumatism; three years later the non-rheumatic cases in the series were again investigated, with the result that the proportion of rheumatic cases rose to 43.5 per cent.; three years later again the previously non-rheumatic remnant was again found to be reduced, the percentage of cases in which rheumatism had declared itself being raised to 53, as against the 32 per cent. of the original investigation six years before.

This tendency for the evidence of a rheumatic diathesis in the case of choreic patients to accumulate with lapse of time is insisted upon by Holt, of New York. His experience has been that if articular symptoms only be accepted as evidence of rheumatism, then 56 per cent. of cases of chorea are rheumatic, while if the cases with endocarditis, but without articular, symptoms be included, as, in his opinion, they ought to be, the proportion of rheumatic cases is still higher. Holt defines chorea as "a functional nervous disease characterised by aimless irregular movements," and concludes his discussion of the ætiology of the malady as follows: "The causes which underlie the occurrence of chorea, therefore, seem to be a rheumatic diathesis, a neurotic constitution, anæmia, and some severe disturbance of general nutrition. . . A very large number of the cases of chorea are in children who present distinct evidences of rheumatism. . . In another group the neurotic element predominates, and in these there may be no connection whatever with rheumatism."

We have to deal, then, with a neurosis, according to these two weighty authorities. Without being able ourselves to formulate a more acceptable hypothesis, we confess to a want of complete satisfaction with this view. If chorea be a neurosis reflecting a debility of the nerve-centres brought about by "the rheumatic diathesis" (which, if it means anything in the light of recent bacteriological research, means a soil favourable to the growth of the diplococcus of rheumatism), if this be so, how comes it that patients who are not, and never have been, choreic, but suffer from rheumatic fever—more particularly those who are suffering from the incidental cardiac disorders of the latter disease—are quite often the very reverse of neurotic? Moreover, why, in the case of the "rheumatic diathesis," should a neurotic manifestation assume with such constancy the strange features of chorea? These and such-like difficulties suggest that chorea and rheumatism have some association more intimate than that with which it is customary to credit them. But at present proof is wanting.

The multitude of remedies for chorea indicate the small success which has attended any particular drug treatment. Indeed, all authors are agreed that there is no such thing as a specific for chorea. Dr. Grenet has collated the opinions of a considerable number of physicians, and has combined them in the following summary of treatment.

Firstly, physical means. In a few mild cases mere hygienic regulation of the daily life is sufficient to achieve a cure, regulation, that is to say, directed towards freedom from worry and excitement. If the case is at all severe absolute rest in bed must be enjoined. Hydrotherapy is strongly praised by some, whether in the form of cold douches, tepid sponge-baths, or the wet pack; Triboulet is strongly opposed to all hydro-therapeutic measures during the active stages of the disease, especially if there is any suspicion of the integrity of the heart. None the less, the author insists upon the value of this therapeutic means when employed with prudence. (We have ourselves observed a rapid onset of pericarditis upon the treatment of a very chronic arthritis of the knee by alternating douches of hot and cold water, an experience corroborative of Triboulet's caution).

Electricity is sometimes useful; best in the shape of a static bath for ten minutes daily, or, failing this, the continuous current applied to the vertebral column.

Some notion of the shifts to which physicians may be driven in the attempt to cure chorea may be gathered from the fact that two Italian physicians have actually made trial of lumbar puncture, and report favourably of it. Dr. Grenet was enterprising enough to try this heroic proceeding, but with no better result than the production in his patients of vomiting and headache.

Dealing with the medicinal treatment of chorea, Dr. Grenet bids us to pin our faith to antipyrine and arsenic.

Antipyrin must be given in large doses, say three grammes to a child of six years, the dose being gradually raised so that six grammes are taken on the third day. If, in spite of these large doses, no good effect has appeared by the third week, it is well to admit that for that particular case the drug is ineffective. Antipyrin is usually well tolerated, but will at times give rise to eruptions upon the skin, and to digestive and urinary troubles.

For the rest we learn nothing more than that arsenic, given as Fowler's solution and in heroic doses, is still the great resource. Dr. Goodhart advises gradual raising of the dose up to 15 minims or more, and Dr. Emmet Holt observes that the index of saturation with arsenic is some disturbance of the stomach or bowels, with puffiness under the eyes, to which we may add the "silvery tongue" not uncommonly seen under circumstances of saturation with arsenic. Dr. Goodhart values sulphate of zinc, Dr. Eustace Smith the extract of ergot, and Holt, for cases definitely associated with rheumatism, the salicylate of soda. There is thus no lack of alternatives, but we fancy the bulk of opinion is on the side of arsenic, rest, and general tonics.

[1] Arch. Gén. de Méd., September 12, 1905.

